# Combinations of medicines in patients with polypharmacy aged 65–100 in primary care: Large variability in risks of adverse drug related and emergency hospital admissions

**DOI:** 10.1371/journal.pone.0281466

**Published:** 2023-02-08

**Authors:** Ali Fahmi, David Wong, Lauren Walker, Iain Buchan, Munir Pirmohamed, Anita Sharma, Harriet Cant, Darren M. Ashcroft, Tjeerd Pieter van Staa

**Affiliations:** 1 Centre for Health Informatics & Health Data Research UK North, Division of Informatics, Imaging and Data Science, School of Health Sciences, Faculty of Biology, Medicine and Health, The University of Manchester, Manchester Academic Health Science Centre, Manchester, United Kingdom; 2 Institute of Population Health, NIHR Applied Research Collaboration North West Coast, University of Liverpool, Liverpool, United Kingdom; 3 Centre for Drug Safety Science, Institute of Systems, Molecular and Integrative Biology (ISMIB) University of Liverpool, Liverpool, United Kingdom; 4 Chadderton South Health Centre, Eaves Lane, Chadderton, United Kingdom; 5 Centre for Pharmacoepidemiology and Drug Safety, NIHR Greater Manchester Patient Safety Translational Research Centre, School of Health Sciences, Faculty of Biology, Medicine and Health, The University of Manchester, Manchester, United Kingdom; Gabriele d’Annunzio University of Chieti and Pescara: Universita degli Studi Gabriele d’Annunzio Chieti Pescara, ITALY

## Abstract

**Background:**

Polypharmacy can be a consequence of overprescribing that is prevalent in older adults with multimorbidity. Polypharmacy can cause adverse reactions and result in hospital admission. This study predicted risks of adverse drug reaction (ADR)-related and emergency hospital admissions by medicine classes.

**Methods:**

We used electronic health record data from general practices of Clinical Practice Research Datalink (CPRD GOLD) and Aurum. Older patients who received at least five medicines were included. Medicines were classified using the British National Formulary sections. Hospital admission cases were propensity-matched to controls by age, sex, and propensity for specific diseases. The matched data were used to develop and validate random forest (RF) models to predict the risk of ADR-related and emergency hospital admissions. Shapley Additive eXplanation (SHAP) values were calculated to explain the predictions.

**Results:**

In total, 89,235 cases with polypharmacy and hospitalised with an ADR-related admission were matched to 443,497 controls. There were over 112,000 different combinations of the 50 medicine classes most implicated in ADR-related hospital admission in the RF models, with the most important medicine classes being loop diuretics, domperidone and/or metoclopramide, medicines for iron-deficiency anaemias and for hypoplastic/haemolytic/renal anaemias, and sulfonamides and/or trimethoprim. The RF models strongly predicted risks of ADR-related and emergency hospital admission. The observed Odds Ratio in the highest RF decile was 7.16 (95% CI 6.65–7.72) in the validation dataset. The C-statistics for ADR-related hospital admissions were 0.58 for age and sex and 0.66 for RF probabilities.

**Conclusions:**

Polypharmacy involves a very large number of different combinations of medicines, with substantial differences in risks of ADR-related and emergency hospital admissions. Although the medicines may not be causally related to increased risks, RF model predictions may be useful in prioritising medication reviews. Simple tools based on few medicine classes may not be effective in identifying high risk patients.

## Introduction

A recent UK Government Review of Overprescribing of medicines highlighted the need to reduce prescribing as at least 10% of the current volume of medicines in the UK may be unnecessary [1, 2]. Older patients frequently receive multiple medicines as they are more likely to have multiple long-term conditions. These conditions often result in multiple medicines being prescribed, or polypharmacy, which is particularly common in the frail older people [2]. Polypharmacy is often intended to reduce the risk of future morbidity and mortality in each of the patient’s specific health conditions. The underlying evidence for drug treatment in patients with multiple long-term conditions is often poor as clinical trials usually focus on single conditions and drugs, excluding, participants with multimorbidity and polypharmacy [3]. A recent policy report proposed a pragmatic approach by classifying polypharmacy into ‘appropriate’ and ‘problematic’. Appropriate polypharmacy was defined as pharmacotherapy that extends life expectancy and improves quality of life. In contrast, problematic polypharmacy concerns pharmacotherapy with an increased risk of drug interactions and adverse drug reactions (ADRs), together with impaired adherence to medication and quality of life for patients [4]. The World Health Organization has highlighted that unsafe medication practices and medication errors are a leading cause of injury and avoidable harm in health care systems across the world [5].

A systematic review of problematic polypharmacy, its burden and the effectiveness of interventions to reduce this found that interventions can reduce problematic polypharmacy but without effect on health outcomes. It concluded that evidence of the extent of problematic polypharmacy in the UK, and what interventions are effective is limited [6]. A possible reason for the limited effectiveness of intervention to optimise prescribing in patients with polypharmacy may be the limited screening tools to identify polypharmacy at higher risk of ADRs. The 2015 NICE Medicines optimisation guideline provide general advice on e.g., systems for reporting ADRs but with only limited information on what medicine combinations would need medicine review. It recommended to use screening tools such as STOPP/START, based on pharmacological considerations and expert consensus, to identify potentially inappropriate prescribing and treatments that might be changed [7]. However, a cluster randomised trial found that a structured medicine review based on the STOPP/START criteria reduced prescribing but without any effect on drug-related hospital admissions which was the primary outcome [8]. A recent review found limited evidence that interventions in polypharmacy, such as medication reviews, resulted in clinically significant improvements [6].

The aim of this study was to develop and test a new screening tool for identifying medicine combinations in patients with polypharmacy at high risk of hospital admissions. The approach in this study was data-driven without prior hypotheses of pharmacological plausibility of the effects of the medicines considered.

## Materials and methods

### Database

Data sources were the Clinical Practice Research Databank (CPRD GOLD) [9] and Aurum [10]. CPRD GOLD and Aurum contain longitudinal, anonymised, patient level electronic health records (EHRs) from general practices in the UK. Almost all UK residents are registered with a general practice, which typically provides most of the primary healthcare. If a patient received emergency care (e.g., at Accident & Emergency department) or inpatient or outpatient hospital care, the general practice of the patient will be informed. All UK general practices use EHRs which are provided by different EHR vendors, including EMIS and Vision. EMIS is the most frequently used primary care EHR, whereas Vision used to be used more frequently previously [11]. The CPRD GOLD databases includes general practices that use Vision EHR software system, while Aurum practices use EMIS Web. Practices can change their EHR software although this will be reflected in the start and end of data collection for each practice. CPRD GOLD includes data on about 11.3 million patients [9] and Aurum 19 million patients [10], although practices and patients may have contributed data for varying durations of time. These databases include the clinical diagnoses, medication prescribed, vaccination history, diagnoses, lifestyle information, clinical referrals, as well as patient’s age, sex, ethnicity, smoking history, and body mass index (BMI). The patient-level data from the general practices in England were linked through a trusted third party to hospital admission data (hospital episode statistics) using unique patient identifiers [9]. The hospital data contained information on the date of hospital admission and the clinical diagnoses established at and during admission and coded using ICD-10. Also, linked data were available, starting April 1, 2007 for visits to emergency departments, including the visit day, but presenting diagnosis data was less complete for these visits. Patient-level socioeconomic information was approximated from Index of Multiple Deprivation (IMD) linked to the patient’s residential postcode [12]. Patient-level IMD was aggregated into quintiles for the current analysis. Medicines were classified using the British National Formulary (BNF) sections which is the prescribing guide for UK clinicians.

### Study population

The overall study population consisted of patients aged 65–100 years at any time during the observation period (from January 1, 2000 to July 1, 2020 for CPRD GOLD or up to September 1, 2020 for Aurum) and registered in a practice from England and participated in record linkage. Patient demographics included sex, age, ethnicity, and medical history. We calculated the Charlson comorbidity score for each patient using their medical history [13]. Follow-up of individual patients considered their start date of registration with a general practice, prior history of registration in the practice of at least three years, time of reaching age 65 as well as end date due to moving away or death and time of reaching age 101. The follow-up of each patient was divided into 3-month periods with risk factors such as presence of morbidity assessed at each of these time-periods. These data were used in the matching process. Presence of polypharmacy, defined as the prescription of ≥ 5 medicines in the 84 days before [2], was assessed at each interval. Most prescriptions are typically issued for a duration of 1–2 months (the 95^th^ percentile of prescription duration was 60 days). Prescribing in the 84 days before the start of each interval was assessed and the number of distinct drug classes counted. Non-pharmacological prescribing, such as blood glucose monitoring equipment, dressings, stoma, or urinary catheter-related products and vaccines, was not included.

The outcomes of interest were based on hospital admission data from the linked data. Two sets of hospital admissions were analysed in this study, including (i) admission code for an adverse-drug reaction (ADR) and (ii) emergency hospital admission. For ADR-related hospital admission, we used a code list based on a systematic search and assessment of lists in 41 publications identifying ADRs from administrative data [14]. This review suggested a comprehensive list of definitions and their corresponding codes, classifying them according to level of likely causality based on the ICD-10 code, which could be used to build consensus among health researchers [14]. The categories used in the current study included (i) ICD-10 codes with phrase ‘induced by medication/drug’, (ii) ICD-10 codes with phrase ‘induced by medication or other causes’ or ‘poisoning by medication’, (iii) ADRs deemed to be very likely or (iv) likely although the ICD-10 code description does not refer to a drug [14]. Emergency hospital admissions were defined as hospital admissions with a visit to the Accident & Emergency on the same day as the hospital admission (following the approach by Budnitz et al. [15]).

Cases were patients with a first hospital admission during follow-up and with recent history of polypharmacy. Cases were matched to up to six controls without hospital admission on the index date (hospital admission date of case) and with history of polypharmacy. The objective of the matching was to closely match on extent of morbidity based on disease (although not on treatments). Matching was done using propensity matching (using the QAdmission Score) as well as matching by variables including age, sex, morbidity cluster, presence of frailty, practice coding level and calendar time. The QAdmissions score estimates the risk of emergency hospital admission for patients aged 18–100 years in primary care [16]. It is based on variables such as age, sex, deprivation score, ethnicity, lifestyle variables (smoking, alcohol intake) and chronic diseases [16]. Predictors such as prescribed medications and laboratory values were not used in the calculation as medications were the exposure of interest and laboratory values were not extracted. Age and calendar time matching was done stepwise (age same year or birth up to difference of up to five years; calendar time from within three months up to difference up to five years). Larger clusters of co-morbidity were also identified using k-means methods. Using 38 conditions [17], the number of clusters was increased stepwise until the number of patients in smaller clusters exceeded 5% of the size of the population. For each practice, the mean level of coding was assessed for each general practice. Nine inception cohorts of starters of medications were identified (including antiarrhythmics, drugs for hypertension / heart failure, thyroid disorders, anti-Parkinson drugs, anti-dementia drugs, antidepressants, antiepileptics, antihyperglycemic therapy and inhaled bronchodilators). The presence of a code for the indication of treatment was measured and then averaged across the practice. Cases and controls were matched on the quintile of practice coding level (mean in CPRD of 64.6% with 5–95% range of 54.4 to 76.6; Aurum 74.4%, 61.6–85.7%). Matching was done separately for CPRD GOLD and Aurum and the risk-set approach to control sampling was used (with control patients potentially included as controls for multiple cases although only once for a particular case).

### Statistical analysis

The propensity matching procedure used a caliper (pre-specified maximum difference) of 0.25 of the logit of the propensity score [18]. Greedy nearest neighbour matching was used to select the control unit nearest to each treated unit. The SAS procedure PSMATCH was used to conduct the matching.

Random forest (RF) models were used to predict the probabilities of being a case or control based on the subgroups of medicine classes. RF is a supervised tree-based classifier developed by Breiman [19]. It has been broadly used and cited in different areas including medicine and pharmaceutical applications [20, 21]. Tree-based methods such as RF offer superior performance for sub-group classification over techniques such as logistic regression due to its difficulty to a-priori define the subgroups [22]. The RF method first creates subsets of the original data by sampling with replacement on the rows of the original data and randomly selecting the features or columns of the original data. This process is known as bootstrapping. After this, RF forms an ensemble of trees that are trained by each subset of the data independent from other trees. The prediction of each tree depends on a randomly chosen vector and produces a random vector of θ independently [20]. This leads to generation of a set of random classifiers that are generalised. For classification with RF, a number of parameters need to be specified including the number of trees in the forest, the maximum depth of the tree, and the maximum number of leaf nodes [19, 23]. To explain RF models, we used SHapley Additive eXplanation (SHAP) values, that can explain the role of each feature or predictor variable in making prediction [24]. SHAP values are calculated by removing each feature and measuring its marginal contribution. They can explain the output of the model as a global interpretability of feature importance, impact of top features toward target prediction (i.e., ADR-related and emergency hospital admissions), and local interpretability of the prediction of a single observation (i.e., one patient). Global interpretability is drawn as feature importance plots that rank the features in a descending order based on the average impact of each feature on model output calculated as the mean of absolute SHAP value of the features. The impact of top features is depicted by ranking the features along with the impact of individual observations on each feature for prediction of the target variable. In this depiction of feature importance, each observation is represented by a dot and the horizontal location of the dots indicates whether the variable’s observations associate with the risk for the target variable or not. The baseline shows no impact on predictions and the farther from the baseline to the right side refers to a greater risk for the target variable. Local interpretability demonstrates the role of each feature on the prediction of one specific observation [25]. This type of explanation specifies a base value that points the base prediction of the model in the absence of any features [26].

The study population was split into a development (75%) and validation (25%) datasets. The first step in the development of the RF models was to select the top 50 medicine classes based on the variable importance in the models. The second step was to estimate the probabilities of being a case or control for these top 50 medicine classes. The reason was that RF models would not converge, due to memory constraints, with detailed RF estimations for the probabilities. Two types of plots explain the prediction of RF models for ADR-related hospital admissions and emergency hospital admissions. These plots express the contribution of each medicine class on hospital admissions with colour-encoding to differentiate cases and controls.

The propensity matching was done using SAS software version 9.4; the RF analyses were done with Python 3.7 using Jupyter Notebooks, although they were redone using SAS with high correlations found between the two packages. We used SHAP package to explain the prediction of RF models for hospital admission predictions [27].

## Results

89,235 cases with polypharmacy and hospitalised with an ADR-related admission were matched to 443,497 controls on age, sex and disease characteristics. A small number of cases (1.1%) could not be matched to any control and were excluded. Most cases were matched by year of birth and within 3 months (81.1%). Table 1 shows characteristics of cases and controls stratified by Aurum and CPRD GOLD. The age and sex distributions were similar between cases and controls (due to the matching). Comparing medical history between cases and one randomly sampled control (per case) showed that medical histories were broadly comparable. Older cases were found to have fewer controls than younger cases. S1 Table provides characteristics of cases of emergency hospital admissions and their matched controls. We found over 112,000 different combinations of the 50 BNF categories that were most important in predicting ADR-related hospital admission in the RF models. For emergency hospital admissions, there were over 484,000 combinations.

**Table 1:** Characteristics of cases with ADR-related hospital admissions and matched controls stratified by data source.

	CPRD GOLD			Aurum		
	Cases	Controls	One control per case	Cases	Controls	One control per case
	(N = 14435)	(N = 58039)	(N = 14435)	(N = 74800)	(N = 385458)	(N = 74800)
Sex women (%)	8473 (58.7%)	35652 (61.4%)	8473 (58.7%)	42284 (56.5%)	223389 (58%)	42284 (56.5%)
Age mean (SD)	79.0 (8.0)	78.1 (7.8)	79.0 (8.0)	79.0 (8.0)	78.6 (7.8)	79.0 (7.9)
Ethnicity						
Caucasian	13631 (94.4%)	53106 (91.5%)	13224 (91.6%)	69362 (92.7%)	351313 (91.1%)	68257 (91.3%)
Unknown	299 (2.1%)	2808 (4.8%)	675 (4.7%)	1587 (2.1%)	15637 (4.1%)	2935 (3.9%)
Charlson score						
1-Very Low	2392 (16.6%)	14617 (25.2%)	2869 (19.9%)	11788 (15.8%)	81174 (21.1%)	13973 (18.7%)
2	5429 (37.6%)	25304 (43.6%)	6147 (42.6%)	26606 (35.6%)	158285 (41.1%)	29691 (39.7%)
3	4236 (29.3%)	13065 (22.5%)	3651 (25.3%)	21233 (28.4%)	96384 (25%)	19588 (26.2%)
4	1726 (12.0%)	4038 (7.0%)	1332 (9.2%)	10511 (14.1%)	36590 (9.5%)	8257 (11%)
5-Very High	652 (4.5%)	1015 (1.7%)	436 (3%)	4662 (6.2%)	13025 (3.4%)	3291 (4.4%)
Risk score for hospital admissions (mean)	17.6 (11.3)	14.7 (9.4)	17.3 (11.1)	17.6 (11.7)	15.9 (10.4)	17.4 (11.6)
Risk score for mortality (mean)	9.8 (10.0)	7.5 (8.3)	9.5 (10.0)	11 (11.1)	9.5 (9.8)	10.7 (10.9)
Medical history						
Atrial fibrillation	2290 (15.9%)	6794 (11.7%)	2345 (16.2%)	13459 (18%)	64369 (16.7%)	14131 (18.9%)
Congestive heart failure	1771 (12.3%)	4186 (7.2%)	1568 (10.9%)	10839 (14.5%)	42047 (10.9%)	9894 (13.2%)
Cancer	808 (5.6%)	2351 (4.1%)	967 (6.7%)	5840 (7.8%)	28397 (7.4%)	7344 (9.8%)
Asthma/chronic obstructive lung disease	2799 (19.4%)	9936 (17.1%)	2982 (20.7%)	15905 (21.3%)	79550 (20.6%)	16976 (22.7%)
Cardiovascular disease	5804 (40.2%)	20343 (35.1%)	5870 (40.7%)	30690 (41%)	150237 (39%)	31309 (41.9%)
Diabetes mellitus type 2	4022 (27.9%)	13968 (24.1%)	3740 (25.9%)	21826 (29.2%)	101554 (26.3%)	20527 (27.4%)
Dementia	971 (6.7%)	3114 (5.4%)	997 (6.9%)	4368 (5.8%)	19356 (5%)	4143 (5.5%)

The calibration of the RF probabilities in the development and validation datasets is shown in Table 2. The RF probabilities were strongly predictive of risk of ADR-related and emergency hospital admission. The observed Odds Ratio (OR) in the highest RF decile was 7.16 (95% CI 6.65–7.72) in the validation dataset, compared to the lowest decile. The RF probabilities of being a case were close to the observed probabilities. The ORs as predicted by RF were smaller than the observed OR in the highest deciles (a small change in the probabilities can lead to substantive difference in the OR in case of higher probabilities).

**Table 2:** Observed and predicted ORs of ADR-related and emergency hospital admissions stratified by deciles of predicted probability of being a case.

Decile	Development				Validation			
	Predicted probability of being case	Observed probability of being case	Predicted OR	Observed OR (95% CI)	Predicted probability of being case	Observed probability of being case	Predicted OR	Observed OR (95% CI)
ADR-related hospital admission								
1	0.08	0.08	reference	reference	0.08	0.08	reference	reference
2	0.10	0.09	1.06	1.29 (1.23–1.36)	0.10	0.09	1.06	1.30 (1.19–1.41)
3	0.11	0.10	1.15	1.35 (1.29–1.42)	0.11	0.10	1.15	1.49 (1.36–1.62)
4	0.12	0.12	1.43	1.55 (1.48–1.62)	0.12	0.12	1.43	1.68 (1.55–1.82)
5	0.13	0.13	1.59	1.65 (1.57–1.73)	0.13	0.13	1.59	1.83 (1.69–1.99)
6	0.15	0.15	1.82	2.01 (1.92–2.11)	0.14	0.15	1.82	2.09 (1.93–2.27)
7	0.18	0.18	2.10	2.51 (2.40–2.62)	0.18	0.18	2.10	2.75 (2.55–2.98)
8	0.20	0.22	2.61	2.93 (2.80–3.06)	0.20	0.22	2.60	3.05 (2.82–3.29)
9	0.24	0.26	3.14	3.77 (3.61–3.93)	0.24	0.26	3.11	4.02 (3.73–4.34)
10	0.37	0.35	4.21	6.90 (6.62–7.20)	0.37	0.35	4.18	7.16 (6.65–7.72)
Emergency hospital admission								
1	0.10	0.09	reference	reference	0.10	0.09	reference	reference
2	0.11	0.10	1.10	1.20 (1.18–1.22)	0.11	0.10	1.10	1.18 (1.15–1.22)
3	0.12	0.12	1.30	1.36 (1.34–1.38)	0.12	0.12	1.30	1.35 (1.31–1.39)
4	0.14	0.13	1.42	1.60 (1.57–1.63)	0.14	0.13	1.42	1.55 (1.50–1.60)
5	0.15	0.15	1.58	1.68 (1.65–1.71)	0.14	0.15	1.59	1.62 (1.58–1.67)
6	0.16	0.16	1.72	1.81 (1.78–1.84)	0.16	0.16	1.73	1.83 (1.77–1.88)
7	0.17	0.18	1.88	2.01 (1.98–2.05)	0.17	0.18	1.89	1.96 (1.91–2.02)
8	0.19	0.20	2.10	2.29 (2.25–2.33)	0.20	0.20	2.11	2.30 (2.24–2.37)
9	0.22	0.23	2.44	2.76 (2.72–2.81)	0.22	0.23	2.47	2.76 (2.69–2.84)
10	0.30	0.29	3.05	4.05 (3.99–4.11)	0.30	0.29	3.09	4.06 (3.95–4.17)

Table 3 gives the discrimination of different logistic models for ADR-related and emergency hospital admissions. The effects of age/sex, Qadmission score and RF scores on the C-statistic were moderate for each of these individually. The C-statistics for ADR-related hospital admissions were 0.58 for age and sex and 0.66 for RF probabilities.

**Table 3:** Discrimination of different logistic models for ADR-related and emergency hospital admissions.

Outcome	Model	C statistic
ADR-related hospital admission	Age and sex only	0.58
	Age, sex and disease characteristics	0.63
	Qadmission score (without prescribed medications and laboratory values)	0.61
	RF probabilities (in development set of cases and controls matched by age, sex and disease characteristics)	0.67
	RF probabilities (in validation set of cases and controls matched by age, sex and disease characteristics)	0.66
Emergency hospital admission	Age and sex only	0.62
	Age, sex and disease characteristics	0.65
	Qadmission score (without prescribed medications and laboratory values)	0.65
	RF probabilities (in development set of cases and controls matched by age, sex and disease characteristics)	0.63
	RF probabilities (in validation set of cases and controls matched by age, sex and disease characteristics)	0.62

**Fig 1:**
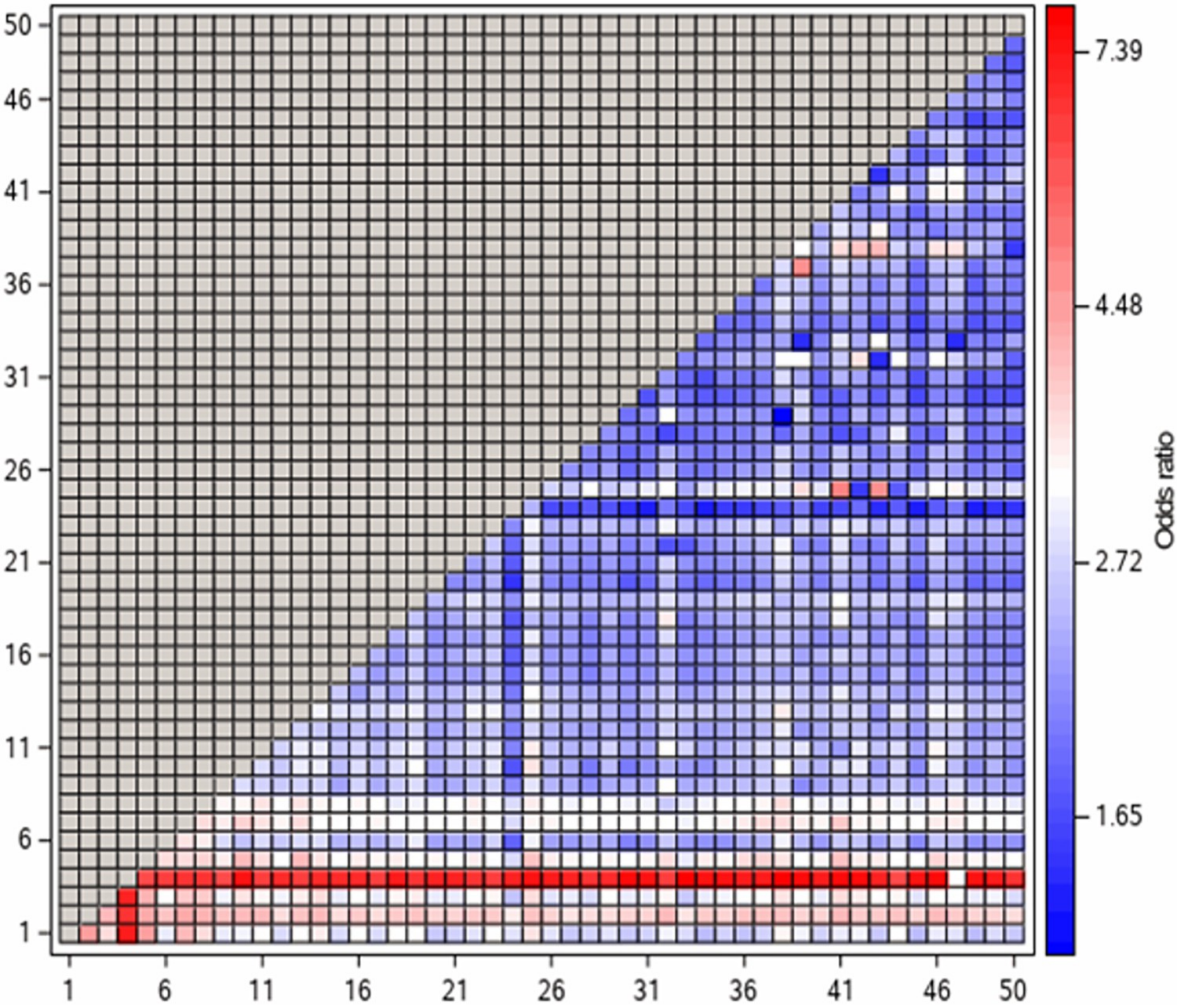
Heatmap of ORs of ADR-related hospital admission in patients using combinations of least two medicine classes, i.e., mean predicted probability of being a case with each combination compared to the 5^th^ percentile of predicted probability. Decodes for the number of each medicine class are provided in Table 4).

Fig 1 shows a heatmap of ORs of ADR-related hospital admission in patients prescribed combinations of at least two medicine classes. For most medicine classes, there was substantive variations in the ORs depending on the co-medication. S1 Fig shows similar results for emergency hospital admissions.

**Table 4:** Range of ORs for ADR-related hospital admission for various medicine classes based on predictions by random forest models (medicine classes ranked in descending order by variable importance in the random forest models).

Number	Medicine class	Range of ORs in users of medicine class^#^		
		OR 2.5^th^ percentile	OR 50^th^ percentile	OR 97.5^th^ percentile
1	Loop diuretics	1.63	2.36	4.85
2	Domperidone and/or metoclopramide	2.88	3.50	5.32
3	Iron-deficiency anaemias	2.11	2.76	5.04
4	Hypoplastic, haemolytic and renal anaemias	5.68	7.47	10.68
5	Sulfonamides and/or trimethoprim	2.31	2.91	5.41
6	Opioid analgesics	1.33	1.94	4.45
7	Quinolones	2.18	2.91	5.18
8	Metronidazole, tinidazole and/or ornidazole	2.06	2.79	4.95
9	Antipsychotic drugs (including typical and atypical)	1.55	2.08	4.41
10	Gout and cytotoxic induced hyperuricemia	1.13	2.14	4.81
11	Drugs for nausea or vertigo: antihistamines	1.20	2.11	4.80
12	Antispasmodics	1.57	2.12	4.20
13	Potassium-sparing diuretics and/or aldosterone antagonists	1.28	2.42	4.89
14	Penicillins	1.35	2.02	4.72
15	Other antidepressant drugs (e.g. mirtazapine, duloxetine, venlafaxine)	1.42	1.87	4.17
16	Systematic corticosteroids	1.26	1.81	4.45
17	Selective serotonin re-uptake inhibitors	1.38	1.82	4.25
18	Macrolides	1.06	1.90	4.57
19	Cephalosporins and/or other beta-lactams	1.40	2.34	4.78
20	Non-opioid analgesics and compound preparations	1.02	1.63	4.20
21	Hypnotics	1.21	1.80	4.27
22	Peripheral and central neuropathic pain (pregabalin)	1.07	1.85	4.45
23	Urinary-tract infections (nitrofurantoin and/or methenamine)	1.33	2.23	4.75
24	Thiazides and related diuretics	0.98	1.28	3.32
25	Some other antibacterials (e.g. chloramphenicol, sodium fusidate, colistin)	1.39	2.55	5.29
26	Drugs used in megaloblastic anaemias (hydroxocobalamin, cyanocobalamin, folic acid)	1.05	1.75	4.34
27	H2-receptor antagonists	1.21	1.68	4.20
28	Drugs used for mania and hypomania	1.09	1.77	3.72
29	Anxiolytics	1.05	1.80	4.25
30	Non-steroidal anti-inflammatory drugs	1.02	1.48	3.77
31	Alpha-adrenoceptor blocking drugs	0.99	1.46	4.08
32	Oestrogens in malignant disease	1.25	1.82	4.81
33	Replacement therapy (hydrocortisone and/or fludrocortisone)	1.09	1.67	3.78
34	Renin-angiotensin system drugs	0.98	1.45	4.04
35	Antimalarials (e.g. quinine)	1.02	1.64	4.34
36	Nitrates	1.00	1.63	4.41
37	Other antianginal drugs (e.g. ivabradine, nicorandil, ranolazine)	1.01	1.69	4.55
38	Clindamycin and/or lincomycin	1.02	2.36	4.75
39	Corticosteroids and other immunosuppressants	1.06	1.78	5.53
40	Control of epilepsy	1.03	1.65	4.26
41	Vasodilator antihypertensive drugs	1.02	2.16	4.90
42	Polyene antifungals	1.03	1.93	4.77
43	Centrally-acting antihypertensive drugs	0.98	1.59	4.28
44	Triazole antifungals	1.02	1.84	4.70
45	Statins	0.98	1.41	3.90
46	Treatment of hypoglycaemia (e.g. glocose gel, fructose, diazoxide)	0.99	1.84	4.87
47	Parenteral anticoagulants (e.g. standard and low molecular weight heparins, heparinoids)	1.04	1.93	4.51
48	Beta-adrenoceptor blocking drugs	0.98	1.45	4.03
49	Antihistamines	1.00	1.56	4.23
50	Aminosalicylates	1.01	1.47	3.87

^#^ORs based on the RF probabilities with the medicine class compared to the 5^th^ percentile of the probabilities in the study population.

S2 and S4 Figs display the feature importance of prediction for ADR-related hospital admission and emergency hospital admission, respectively. S3 and S5 Figs show the impact of top features toward the target variables: ADR-related hospital admission and emergency hospital admission, respectively.

Table 4 presents the range of ORs within each medicine class based on RF predictions for ADR-related hospital admissions. These ORs indicate the effect of taking each medicine class compared to not taking the medicine class. The range of ORs (5, 50 and 95^th^ percentiles) provide the variability in the effects depending on co-medication. As an example, the ORs for users of loop diuretics ranged from 1.63 to 4.85. Further details on varying effects of medicine combinations are shown in Table 5 including three levels of medicines based on predictions by RF model. As an example, users of loop diuretics had a mean OR of 7.97 when co-prescribed with medicines for hypoplastic/haemolytic/renal anaemias and clindamycin/lincomycin. Conversely, users of loop diuretics, renin-angiotensin system drugs and beta-adrenoceptor blocking drugs had an OR of 2.53. S2 Table provides the range of ORs within each medicine class for emergency hospital admissions.

**Table 5:** ORs for ADR-related hospital admission for example combinations of medicines based on predictions by random forest model.

Level 1	Level 2	Level 3	Mean OR in each group of users
Loop diuretics			2.54
	Hypoplastic, haemolytic and renal anaemias		7.34
		Clindamycin and/or lincomycin	7.97
		Potassium-sparing diuretics and/or aldosterone antagonists	6.31
	Renin-angiotensin system drugs		2.52
		Hypoplastic, haemolytic and renal anaemias	7.35
		Beta-adrenoceptor blocking drugs	2.53
Domperidone and/or metoclopramide			3.65
	Hypoplastic, haemolytic and renal anaemias		6.64
		Centrally-acting antihypertensive drugs	7.21
		Cephalosporins and/or other beta-lactams	5.74
	Thiazides and related diuretics		3.44
		Hypoplastic, haemolytic and renal anaemias	5.97
		Replacement therapy (hydrocortisone and/or fludrocortisone)	2.89
Iron-deficiency anaemias			2.89
	Hypoplastic, haemolytic and renal anaemias		7.00
		Clindamycin and/or lincomycin	7.97
		Anxiolytics	5.47
	Thiazides and related diuretics		2.59
		Hypoplastic, haemolytic and renal anaemias	6.68
		Replacement therapy (hydrocortisone and/or fludrocortisone)	2.50
Hypoplastic,haemolytic and renal anaemias			7.53
	Replacement therapy (hydrocortisone and/or fludrocortisone)		8.12
		Non-opioid analgesics and compound preparations	8.72
		Iron-deficiency anaemias	7.44
	Triazole antifungals		6.11
		Iron-deficiency anaemias	6.26
		Gout and cytotoxic induced hyperuricemia	5.96
Sulfonamides and/or trimethoprim			3.17
	Hypoplastic, haemolytic and renal anaemias		6.23
		Antispasmodics	7.15
		Antipsychotic drugs (including typical and atypical)	4.84
	Thiazides and related diuretics		2.84
		Loop diuretics	4.55
		Aminosalicylates	2.80
Opioid analgesics			2.10
	Hypoplastic, haemolytic and renal anaemias		6.46
		Some other antibacterials (e.g. chloramphenicol, sodium fusidate, colistin)	7.97
		Anxiolytics	5.47
	Thiazides and related diuretics		1.83
		Hypoplastic, haemolytic and renal anaemias	6.36
		Statins	1.82
Quinolones			3.09
	Hypoplastic, haemolytic and renal anaemias		6.68
		Gout and cytotoxic induced hyperuricemia	7.44
		Antipsychotic drugs (including typical and atypical)	6.00
	Thiazides and related diuretics		2.77
		Domperidone and/or metoclopramide	4.16
		Clindamycin and/or lincomycin	2.24
Metronidazole, tinidazole and/or ornidazole			2.94
	Hypoplastic, haemolytic and renal anaemias		6.60
		Gout and cytotoxic induced hyperuricemia	7.97
		Selective serotonin re-uptake inhibitors	5.48
	Oestrogens in malignant disease		2.66
		Opioid analgesics	2.66
Antipsychotic drugs (including typical and atypical)			2.25
	Hypoplastic, haemolytic and renal anaemias		6.20
		Some other antibacterials (e.g. chloramphenicol, sodium fusidate, colistin)	7.24
		Sulfonamides and/or trimethoprim	4.84
	Corticosteroids and other immunosuppressants		2.09
		Iron-deficiency anaemias	2.89
		Antimalarials (e.g. quinine)	1.74
Gout and cytotoxic induced hyperuricemia			2.13
	Hypoplastic, haemolytic and renal anaemias		7.59
		Corticosteroids and/or other immunosuppressants	8.48
		Peripheral and central neuropathic pain (pregabalin)	5.96
	Thiazides and related diuretics		1.70
		Hypoplastic, haemolytic and renal anaemias	6.68
		Clindamycin and/or lincomycin	1.64

**Fig 2:**
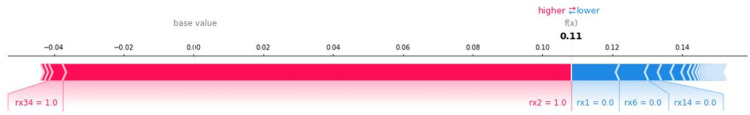
Local interpretation of RF model prediction for ADR-related hospital admissions for fake observation. Decodes for the number of each medicine class are provided in Table 4.

Fig 2 displays a local interpretability of RF model prediction for ADR-related admission for a fake observation. The figure shows that exposure to loop diuretics (rx1), medicines for iron-deficiency anaemias (rx3), opioid analgesics (rx6) and antispasmodics (rx12) was associated with an increased risk of ADR-related hospital admission (red lines). The medicines for iron-deficiency anaemias (rx3) contributed relatively most to the increased risk. Conversely, absence of penicillins (rx14) was associated with a lowered risk (blue lines).

## Discussion

Our study found that primary care patients with polypharmacy were prescribed a myriad combination of medicines. The risks of ADR-related and emergency hospital admissions varied substantially with the specific combinations of medicines. RF models identified sub-groups of medicine users with substantially increased risks of hospital admission (ORs of about 7 for highest vs lowest decile). Loop diuretics, domperidone and/or metoclopramide, medicines for iron-deficiency anaemias and for hypoplastic/haemolytic/renal anaemias, and sulfonamides/trimethoprim were the top 5 medicine classes with highest importance in the RF models for ADR-related and emergency hospital admissions. Various classes of antibiotics (including widely used penicillin, macrolides, cephalosporins, nitrofurantoin and methenamine) were also associated with substantively increased risk of ADR-related and emergency hospital admissions. Medicine classes for pain treatment (such as opioid analgesics and non-opioid analgesics and compound preparations) showed an association with higher risk of ADR-related and emergency hospital admission. Although some analgesics may not be even effective [28], they are usually prescribed to treat chronic pain that older people are more likely to suffer from, which can lead to or exacerbate polypharmacy and its risks [29, 30].

The evidence base for the safety and effectiveness of medicine combinations is limited, and this study has shown this is likely to be a substantial problem for delivering safer care. As outlined in a recent review, older people remain under-represented in clinical trials, and differential effects of medicines under-researched [31]. Treatment guidelines are often developed with a focus on patients with single conditions, and less consideration of multimorbidity and effects of polypharmacy. A review and expert consensus of guidelines for the management of patients with multimorbidity and polypharmacy concluded that there is limited availability of reliable risk prediction models and absence of interventions of proven effectiveness [32]. Despite the widely recognised need for medicine optimisation [5, 33], there are only limited tools available to guide clinicians. A 2015 national guideline in England for medicine optimisation mostly provides general guidance on systems rather than specific patient- or medicine characteristics to act on [34]. One exception is the recommendation to use a screening tool such as STOPP/START tool, which includes 80 STOPP criteria of stopping a medicine or reducing the dose mostly for single disease-medicine or for two medicine combinations [7]. The advantages of the START/STOPP are the detailed considerations by an expert panel of expert and biological plausibility of adverse effects. A major disadvantage is that these sets of criteria do not capture the huge number of medicine combinations with substantive variations in risks in patients with polypharmacy, as observed in our study or acknowledged in the Scottish polypharmacy guidance [35]. RF models may be useful to better capture the large and complex heterogeneity in risks and medicine combinations.

Global interpretability of RF models can help to distinguish the medicines on level of association to risks such as ADR-related or emergency hospital admissions. Local interpretability can explain the prediction and relative associations of different medicines to risk for one patient, and they may be useful in supporting medication reviews for individual patients. These techniques may provide information on the relative importance of various predictors on risk; however, they do not provide causally explainable evidence. Explainability has been considered an essential prerequisite for machine learning models such as RF models [36]. A widely used method is to focus on medicines with pharmacologically well-established mechanisms that can lead to ADR, like STOPP/START criteria [7]. A recent trial in patients with polypharmacy found that an intervention applying STARTT/STOPP reduced the prevalence of inappropriate medicine use, but without effect on drug related hospital admission [8]. A challenge for managing ADR risks in this way is that polypharmacy is a complex system [37], with very many medicine combinations and with hugely varying risks, as observed in this study. It has been argued that explainability of AI models may not be essential but rather empirical evaluation of successful implementation and effectiveness [38]. In the case of RF models in polypharmacy, such evaluation could involve highlighting medicines at higher ADR risk to clinicians, with any deprescribing decision considering both patient preferences for the medicine and perceived clinical need.

This study was successful in predicting risks of ADR-related and emergency hospital admissions and it could identify the most important medicine classes that contributed to those risks; however, there are several limitations to this study. A major limitation is residual confounding due to differences in disease severity between various medication combinations despite propensity matching. Cases and controls were broadly matched on presence of disease but not on severity of disease. Like most risk prediction models, the results of this study should not be used for counterfactual risk prediction and causal inference [39]. Therefore, the risk difference between exposed and non-exposed patients cannot be assumed to be the effects of the exposure. A limitation of our study is that we do not provide direct evidence for specific interventions to reduce risks. But our results could support targeting of patients at higher risk for ADR-related or emergency hospital admissions, which could be considered for a structured medication review. Another limitation is that medicines were combined into sometimes broad categories covering various pharmacological effects. A further limitation is that our study focuses on hospital admission of older people; however, there can be other adverse outcomes related to polypharmacy such as losing independence, incontinence, or deteriorating cognition. Also, not only older people, but also younger people with complex multimorbidity and polypharmacy can be the subject of these adverse outcomes and may need a medication review.

In conclusion, polypharmacy involves very large number of different combinations of medicines, with substantial differences in risks of ADR-related and emergency hospital admissions. Although the medicines may not be causally related to increased risks, RF models may be used to target interventions to those individuals at greatest need. Simple tools based on counts of medicines or focussed on few medicine classes may not be effective in identifying high risk patients. Predictions based on RF models may help to prioritise patients for structured medication reviews. Future work could involve developing a clinical decision-support with a user interface for doctors to predict and provide the risk of ADR-related and emergency hospital admissions in polypharmacy.

## Supporting information

S1 Table Characteristics of matched cases of emergency hospital admissions and propensity matched controls. (DOCX)

S2 Table Range of ORs for emergency hospital admission within each medicine class based on predictions by random forest models (ranked by in descending order by variable importance). (DOCX)

S1 Fig Heatmap of ORs of emergency hospital admission in patients using combinations of least two medication classes., i.e., mean predicted probability of being a case with each combination compared to the 5^th^ percentile of predicted probability. Decodes for the number of each medication class is provided in S2 Table.
(TIF)

S2 Fig Feature importance of RF model for ADR-related hospital admissions, ranking of the top 20 features. Decodes for the number of each medication class is provided in Table 4.
(TIF)

S3 Fig Impact of top features of RF model for ADR-related hospital admissions, ranking of the top 20 features along with a summary of individual impacts of observations for each feature. Decodes for the number of each medication class is provided in Table 4.
(TIF)

S4 Fig Feature importance of RF model for emergency hospital admissions, ranking of the top 20 features. Decodes for the number of each medication class is provided in S2 Table.
(TIF)

S5 Fig Impact of top features of RF model for emergency hospital admissions, ranking of the top 20 features along with a summary of individual impacts of observations for each feature on emergency hospital admissions. Decodes for the number of each medication class is provided in S2 Table.
(TIF)
