# Supplementary material for: Combinations of medicines in patients with polypharmacy aged 65–100 in primary care: Large variability in risks of adverse drug related and emergency hospital admissions
Source: PLoS One. 2023 Feb 8;18(2):e0281466. doi: 10.1371/journal.pone.0281466 (PMC9907844; doi:10.1371/journal.pone.0281466)
Supplement: S1 Table — (DOCX) [file pone.0281466.s001.docx]

|  | CPRD GOLD | | | Aurum | | |
| --- | --- | --- | --- | --- | --- | --- |
|  | Cases | Controls | One control per case | Cases | Controls | One control per case |
|  | (N=105494) | (N=425768) | (N=105494) | (N=513017) | (N=2687721) | (N=513017) |
| Sex women (%) | 61664 (58.5%) | 258437 (60.7%) | 61664 (58.5%) | 290924 (56.7%) | 1553211 (57.8%) | 290924 (56.7%) |
| Age mean (SD) | 79.9 (8.3) | 78.8 (8.1) | 79.8 (8.3) | 79.8 (8.2) | 79.4 (8.1) | 79.8 (8.2) |
| Ethnicity | |  |  |  |  |  |
| Caucasian | 100053 (94.8%) | 392156 (92.1%) | 96996 (91.9%) | 481681 (93.9%) | 2475325 (92.1%) | 473959 (92.4%) |
| Unknown | 1337 (1.3%) | 17699 (4.2%) | 4575 (4.3%) | 4951 (1.0%) | 79311 (3.0%) | 14413 (2.8%) |
| Charlson score | |  |  |  |  |  |
| 1 - Very Low | 17700 (16.8%) | 94498 (22.2%) | 19020 (18%) | 81563 (15.9%) | 504467 (18.8%) | 87657 (17.1%) |
| 2 | 42364 (40.2%) | 184705 (43.4%) | 44088 (41.8%) | 197031 (38.4%) | 1091350 (40.6%) | 201470 (39.3%) |
| 3 | 30438 (28.9%) | 107149 (25.2%) | 29151 (27.6%) | 148546 (29%) | 740282 (27.5%) | 146582 (28.6%) |
| 4 | 11215 (10.6%) | 31523 (7.4%) | 10222 (9.7%) | 62178 (12.1%) | 268262 (10%) | 57375 (11.2%) |
| 5 - Very High | 3777 (3.6%) | 7893 (1.9%) | 3013 (2.9%) | 23699 (4.6%) | 83360 (3.1%) | 19933 (3.9%) |
| Risk score for hospital admissions (mean) | 18.4 (11.6) | 15.5 (9.8) | 18 (11.4) | 18 (11.6) | 16.5 (10.4) | 17.8 (11.5) |
| Risk score for mortality (mean) | 10.6 (10.6) | 8.2 (8.8) | 10.2 (10.2) | 11.6 (11.3) | 10.2 (10.0) | 11.3 (11.0) |
| Medical history | |  |  |  |  |  |
| Atrial fibrillation | 18569 (17.6%) | 56957 (13.4%) | 18652 (17.7%) | 95018 (18.5%) | 447340 (16.6%) | 94436 (18.4%) |
| Congestive heart failure | 9681 (9.2%) | 25313 (5.9%) | 9421 (8.9%) | 51963 (10.1%) | 217818 (8.1%) | 50644 (9.9%) |
| Cancer | 6292 (6.0%) | 17042 (4.0%) | 6667 (6.3%) | 43024 (8.4%) | 186349 (6.9%) | 45931 (9.0%) |
| Asthma / chronic obstructive lung disease | 25055 (23.8%) | 86623 (20.3%) | 24884 (23.6%) | 127748 (24.9%) | 599784 (22.3%) | 123482 (24.1%) |
| Cardiovascular disease | 41478 (39.3%) | 146898 (34.5%) | 41709 (39.5%) | 202319 (39.4%) | 999650 (37.2%) | 203109 (39.6%) |
| Diabetes mellitus type 2 | 23996 (22.7%) | 89855 (21.1%) | 23471 (22.2%) | 125949 (24.6%) | 637224 (23.7%) | 124470 (24.3%) |
| Dementia | 11101 (10.5%) | 32292 (7.6%) | 9753 (9.2%) | 46751 (9.1%) | 172897 (6.4%) | 34924 (6.8%) |
