# Supplementary material for: Combinations of medicines in patients with polypharmacy aged 65–100 in primary care: Large variability in risks of adverse drug related and emergency hospital admissions
Source: PLoS One. 2023 Feb 8;18(2):e0281466. doi: 10.1371/journal.pone.0281466 (PMC9907844; doi:10.1371/journal.pone.0281466)
Supplement: S2 Table — (DOCX) [file pone.0281466.s002.docx]

| Number | Medication class | OR^#^ 2.5^th^ percentile | OR^#^ 50^th^ percentile | OR^#^ 97.5^th^ percentile |
| --- | --- | --- | --- | --- |
| 1 | Systemic corticosteroids | 1.48 | 2.12 | 3.27 |
| 2 | Loop diuretics | 1.41 | 1.77 | 3.14 |
| 3 | Opioid analgesics | 1.22 | 1.70 | 3.00 |
| 4 | Macrolides | 1.49 | 2.17 | 3.25 |
| 5 | Penicillins | 1.56 | 2.16 | 3.20 |
| 6 | Sulfonamides and/or trimethoprim | 1.85 | 2.27 | 3.13 |
| 7 | Domperidone and/or metoclopramide | 1.44 | 2.09 | 3.12 |
| 8 | Quinolones | 1.26 | 2.07 | 3.24 |
| 9 | Iron-deficiency anaemias | 1.21 | 1.66 | 2.91 |
| 10 | Selective serotonin re-uptake inhibitors | 1.40 | 1.74 | 2.88 |
| 11 | Antipsychotic drugs (including typical and atypical) | 1.52 | 1.85 | 2.90 |
| 12 | Tetracyclines | 1.10 | 1.88 | 3.17 |
| 13 | Drugs for nausea or vertigo: antihistamines | 1.02 | 1.67 | 3.00 |
| 14 | Urinary-tract infections (nitrofurantoin and/or methenamine) | 1.54 | 2.04 | 2.94 |
| 15 | Peripheral and central neuropathic pain (pregabalin) | 1.05 | 1.65 | 2.97 |
| 16 | Non-opioid analgesics and compound preparations | 1.02 | 1.55 | 2.83 |
| 17 | Statins | 0.96 | 1.38 | 2.69 |
| 18 | Theophylline | 1.02 | 1.84 | 3.25 |
| 19 | Nitrates | 1.20 | 1.55 | 2.85 |
| 20 | Cephalosporins and other beta-lactams | 1.36 | 2.01 | 3.05 |
| 21 | Replacement therapy (hydrocortisone and/or fludrocortisone) | 1.14 | 1.66 | 2.70 |
| 22 | Skeletal muscle relaxants | 1.04 | 1.66 | 2.88 |
| 23 | Control of epilepsy | 1.25 | 1.67 | 2.86 |
| 24 | Drugs for dementia | 1.49 | 1.74 | 2.72 |
| 25 | Anxiolytics | 1.07 | 1.75 | 3.02 |
| 26 | Dopaminergic drugs used in parkinsonism | 1.12 | 1.58 | 2.78 |
| 27 | Other antidepressant drugs (e.g. mirtazapine, duloxetine, venlafaxine) | 1.12 | 1.64 | 2.84 |
| 28 | Metronidazole, tinidazole and/or ornidazole | 1.02 | 1.76 | 3.02 |
| 29 | Thiazides and related diuretics | 0.95 | 1.21 | 2.40 |
| 30 | Parenteral anticoagulants (e.g. standard and low molecular weight heparins, heparinoids) | 1.01 | 1.69 | 3.05 |
| 31 | Other antianginal drugs (e.g. ivabradine, nicorandil, ranolazine) | 1.10 | 1.56 | 2.90 |
| 32 | Hypnotics | 1.04 | 1.62 | 2.87 |
| 33 | Renin-angiotensin system drugs | 0.96 | 1.40 | 2.70 |
| 34 | Polyene antifungals | 1.10 | 1.96 | 3.29 |
| 35 | Triazole antifungals | 1.01 | 1.77 | 3.14 |
| 36 | Calcium-channel blockers | 0.96 | 1.34 | 2.65 |
| 37 | Drugs used in megaloblastic anaemias (hydroxocobalamin, cyanocobalamin, folic acid) | 1.01 | 1.53 | 2.80 |
| 38 | Potassium-sparing diuretics and/or aldosterone antagonists | 1.03 | 1.58 | 2.95 |
| 39 | Antiplatelet drugs | 0.96 | 1.41 | 2.71 |
| 40 | Oral anticoagulants | 0.97 | 1.46 | 2.80 |
| 41 | Antispasmodics | 1.01 | 1.56 | 2.86 |
| 42 | Antimuscarinic drugs used in parkinsonism | 1.05 | 1.69 | 2.74 |
| 43 | Treatment of hypoglycaemia (e.g. glucose gel, fructose, diazoxide) | 0.96 | 1.57 | 2.91 |
| 44 | Drugs affecting gonadotrophins | 0.97 | 1.41 | 2.69 |
| 45 | Hormone antagonists | 0.96 | 1.45 | 2.72 |
| 46 | Oestrogens in malignant disease | 1.01 | 1.54 | 2.97 |
| 47 | Clindamycin and lincomycin | 1.03 | 1.90 | 3.16 |
| 48 | Cardiac glycosides | 1.00 | 1.53 | 2.86 |
| 49 | Drugs for urinary frequency enuresis and incontinence | 1.00 | 1.53 | 2.78 |
| 50 | Tricyclic and related antidepressant drugs | 1.00 | 1.53 | 2.83 |

^#^ORs based on the RF probabilities with the medication class compared to the 5^th^ percentile of the probabilities in the study population.
