# Supplementary figures and images for: Combinations of medicines in patients with polypharmacy aged 65–100 in primary care: Large variability in risks of adverse drug related and emergency hospital admissions
Source: PLoS One. 2023 Feb 8;18(2):e0281466. doi: 10.1371/journal.pone.0281466 (PMC9907844; doi:10.1371/journal.pone.0281466)

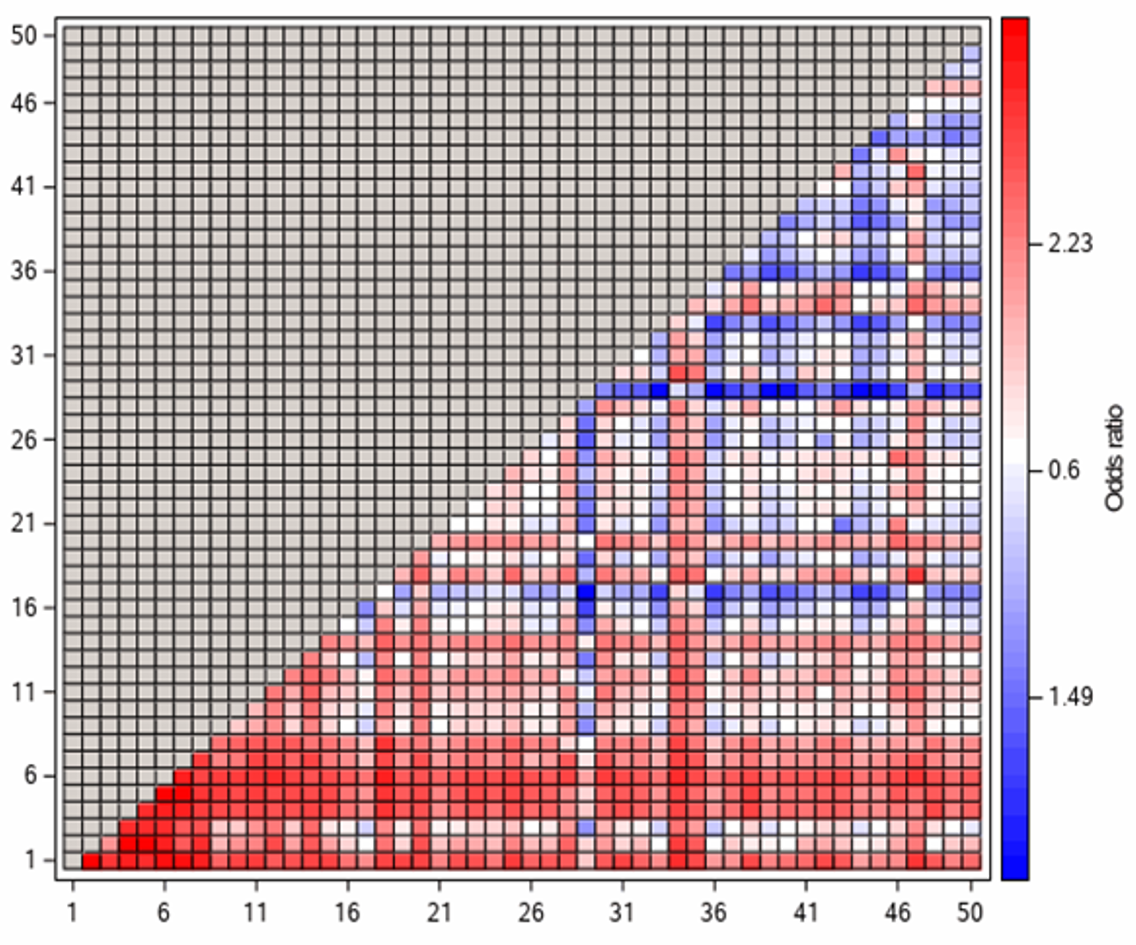

Supplement: S1 Fig — Decodes for the number of each medication class is provided in S2 Table. (TIF) [file pone.0281466.s003.tif]

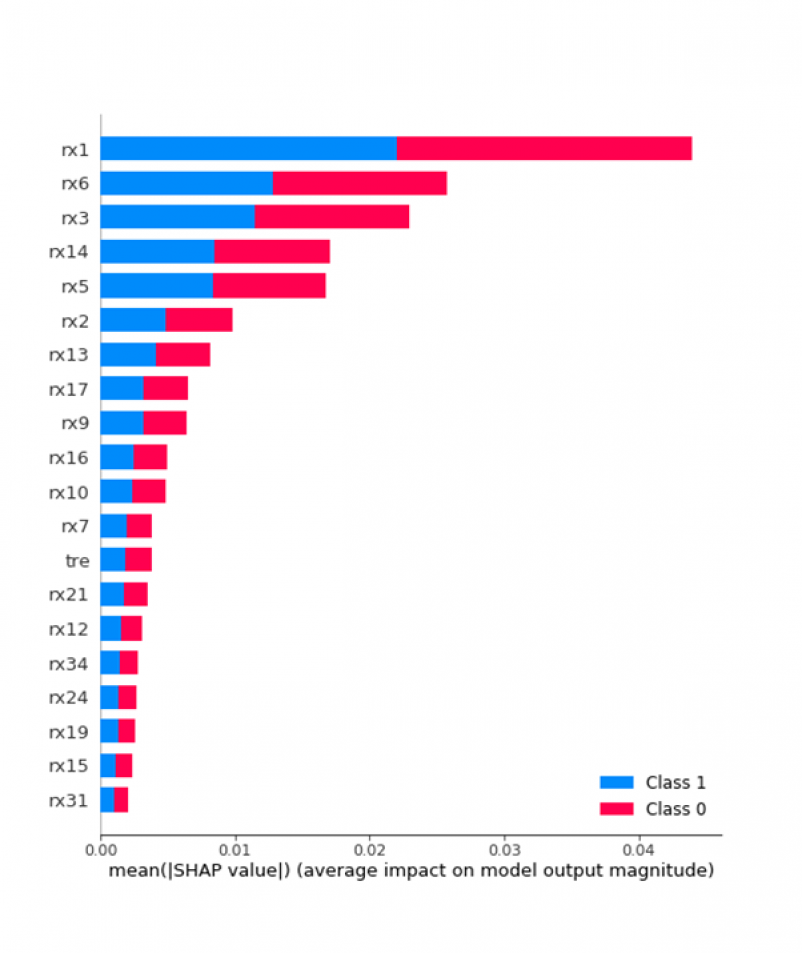

Supplement: S2 Fig — Decodes for the number of each medication class is provided in Table 4. (TIF) [file pone.0281466.s004.tif]

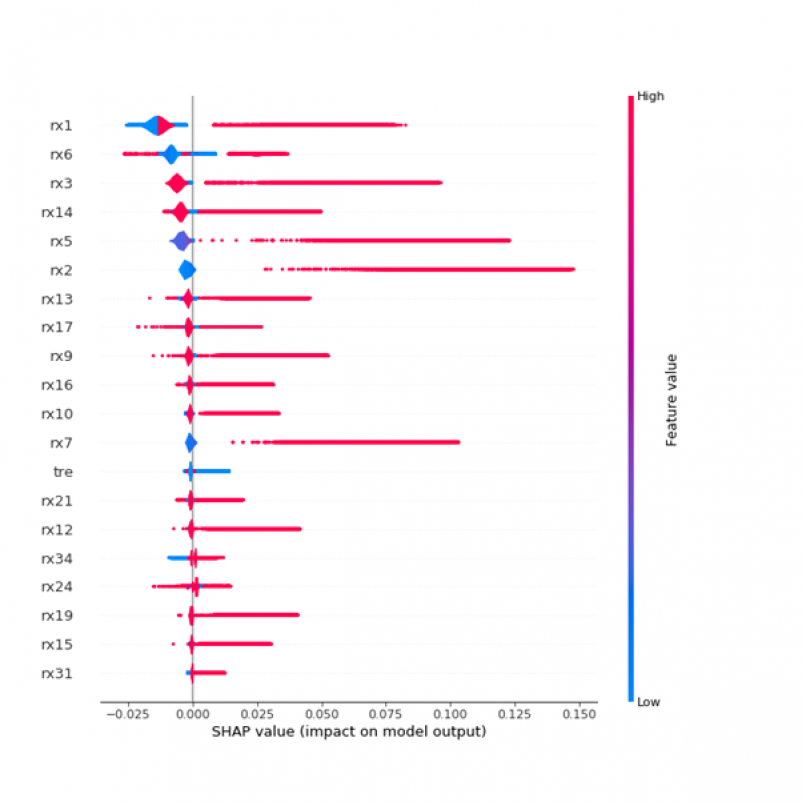

Supplement: S3 Fig — Decodes for the number of each medication class is provided in Table 4. (TIF) [file pone.0281466.s005.tif]

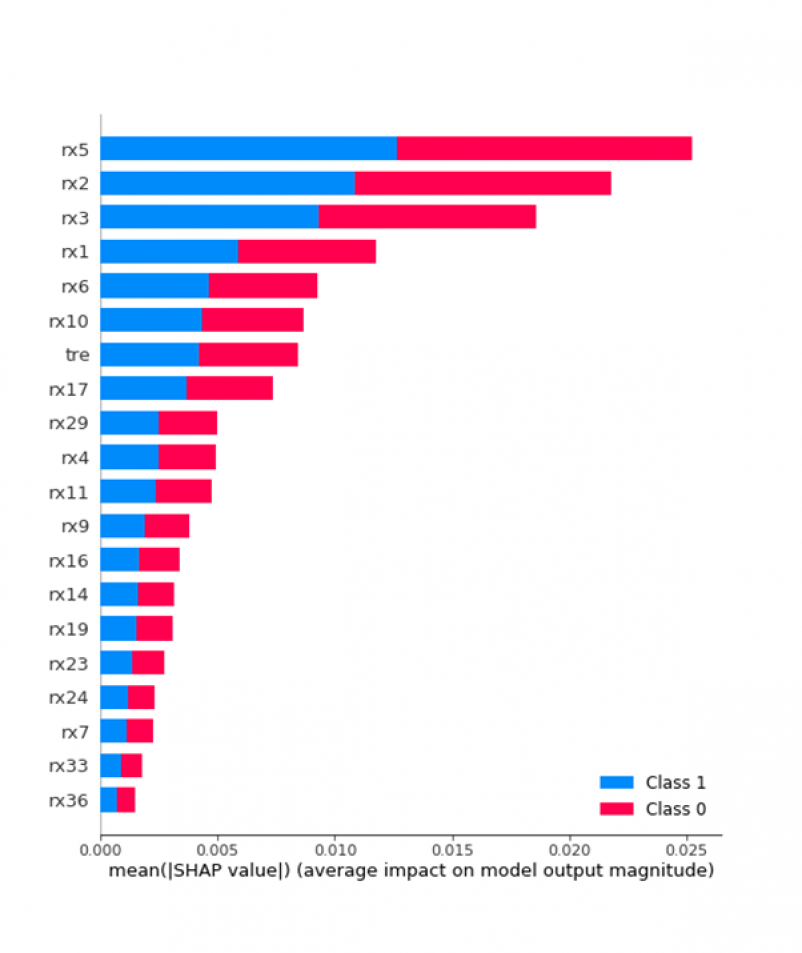

Supplement: S4 Fig — Decodes for the number of each medication class is provided in S2 Table. (TIF) [file pone.0281466.s006.tif]

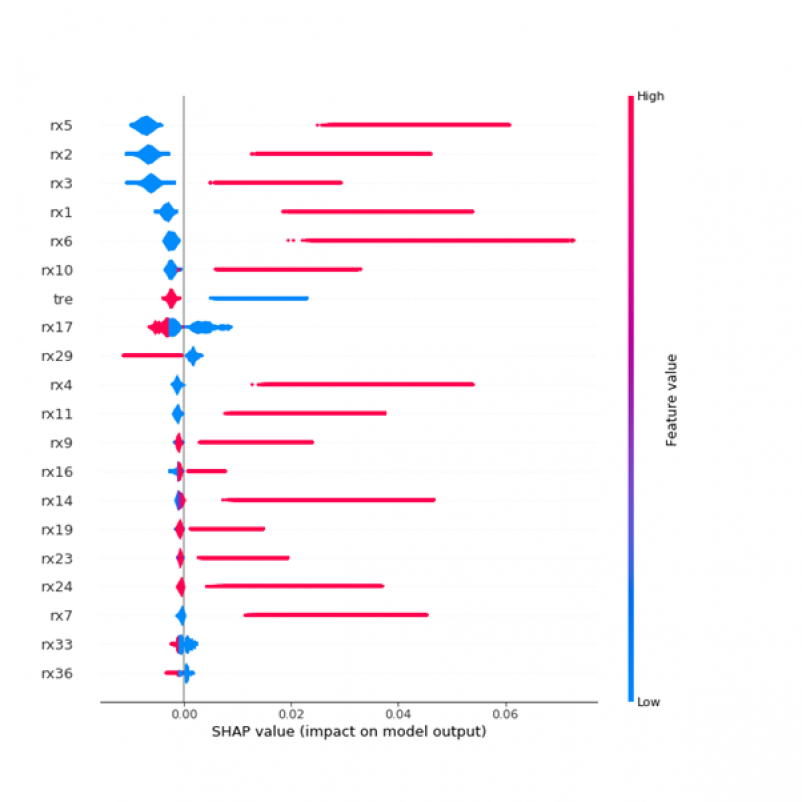

Supplement: S5 Fig — Decodes for the number of each medication class is provided in S2 Table. (TIF) [file pone.0281466.s007.tif]
